# Supplementary material for: Cis-Regulatory Evolution of CCNB1IP1 Driving Gradual Increase of Cortical Size and Folding in primates
Source: bioRxiv. 2024 Dec 9:2024.12.08.627376. Preprint. [Version 1] doi: 10.1101/2024.12.08.627376 (PMC11661109; doi:10.1101/2024.12.08.627376)
Supplement: Supplement 1 [file NIHPP2024.12.08.627376v1-supplement-1.pdf]

bioRxiv preprint doi: https://doi.org/10.1101/2024.12.08.627376; this version posted December 9, 2024. The copyright holder for this preprint (which was not certified by peer review) is the author/funder, who has granted bioRxiv a license to display the preprint in perpetuity. It is made available under aCC-BY 4.0 International license.

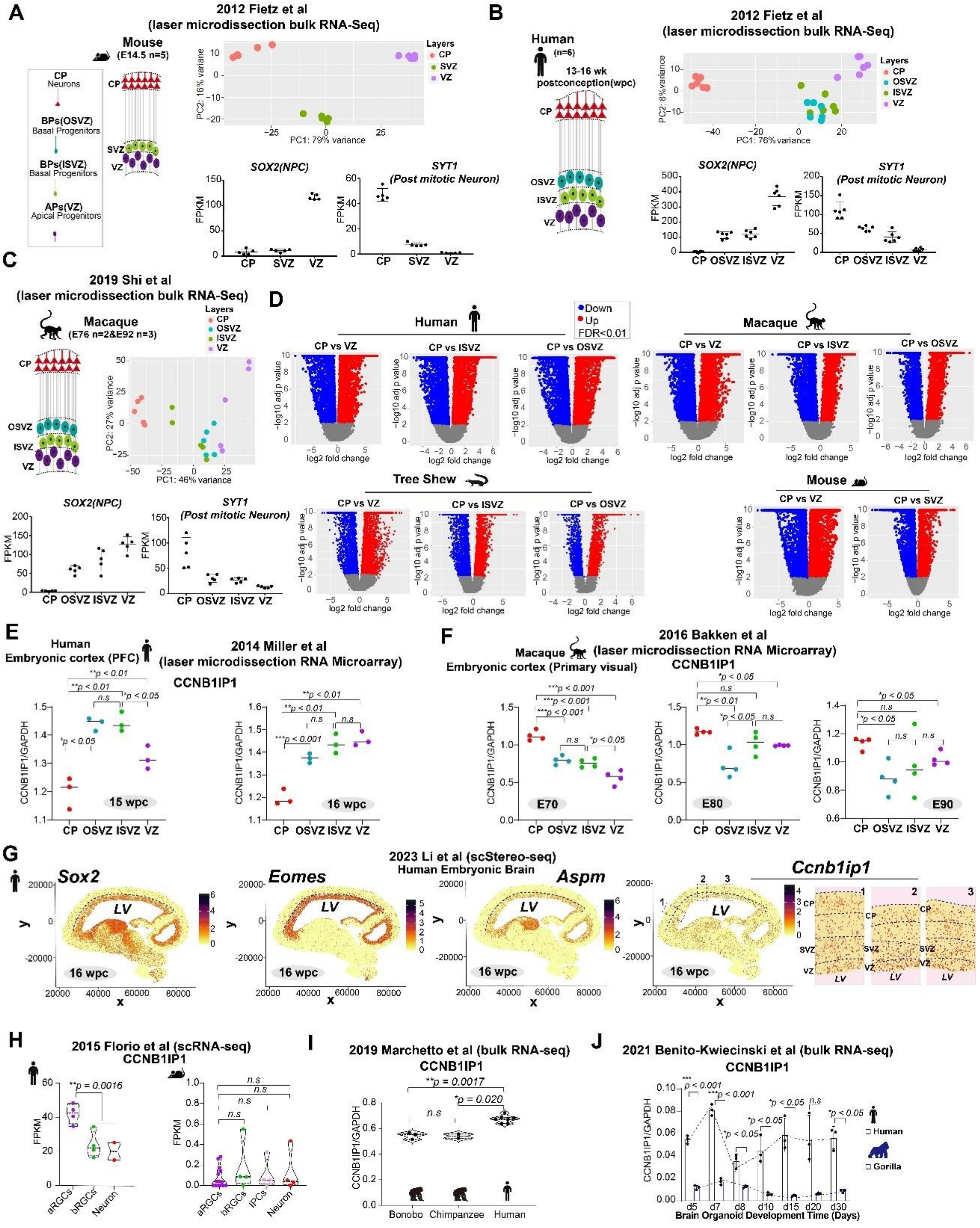

Fig.S1. Transcriptome analysis of human, macaque, Chinese tree shrew, and mouse cortex laminae.

(**A-C**) PCA maps of mice, humans and macaques based on the expression levels of all genes. Bottom panel: cell marker analysis with *SOX2* for neural progenitor cells and *SYT1* for neurons.

bioRxiv preprint doi: https://doi.org/10.1101/2024.12.08.627376; this version posted December 9, 2024. The copyright holder for this preprint (which was not certified by peer review) is the author/funder, who has granted bioRxiv a license to display the preprint in perpetuity. It is made available under aCC-BY 4.0 International license.

(**D**) Volcano plots demonstrating pairwise comparisons of gene expression between the laminae of humans, macaques, Chinese tree shrews, and mice.

(**E**) Scatter plots showing pairwise comparisons of CCNB1I1P expression between the laminae of the human fetal cortex at 15wpc and 16wpc respectively (mean, two-tailed unpaired Student's *t*-test).

(**F**) Scatter plots showing pairwise comparisons of CCNB1I1P expression between the laminae of monkey fetal cortex at embryonic 70, 80 and 90 days (mean, two-tailed unpaired Student's *t*-test).

(**G**) Spatial visualization of SOX2, EOMES, ASPM and CCNB1I1P expression in the human fetal cortex at 16wpc. The spatial visualization data were downloaded from brainAtlas (http://donglab.life/brainAtlas.html).

(**H**) Violin plot showing *CCNB1IP1* expression in human and mouse aRGC, bRGC, and neurons.

(**I**) Violin plot showing *CCNB1IP1* expression in induced pluripotent stem cell (iPS)-derived NPCs among humans, chimpanzees, and bonobos.

(**J**) Bar plot showing *CCNB1IP1* expression during human and gorilla brain organoid development (mean, two-tailed unpaired Student's *t*-test).

bioRxiv preprint doi: https://doi.org/10.1101/2024.12.08.627376; this version posted December 9, 2024. The copyright holder for this preprint (which was not certified by peer review) is the author/funder, who has granted bioRxiv a license to display the preprint in perpetuity. It is made available under aCC-BY 4.0 International license.

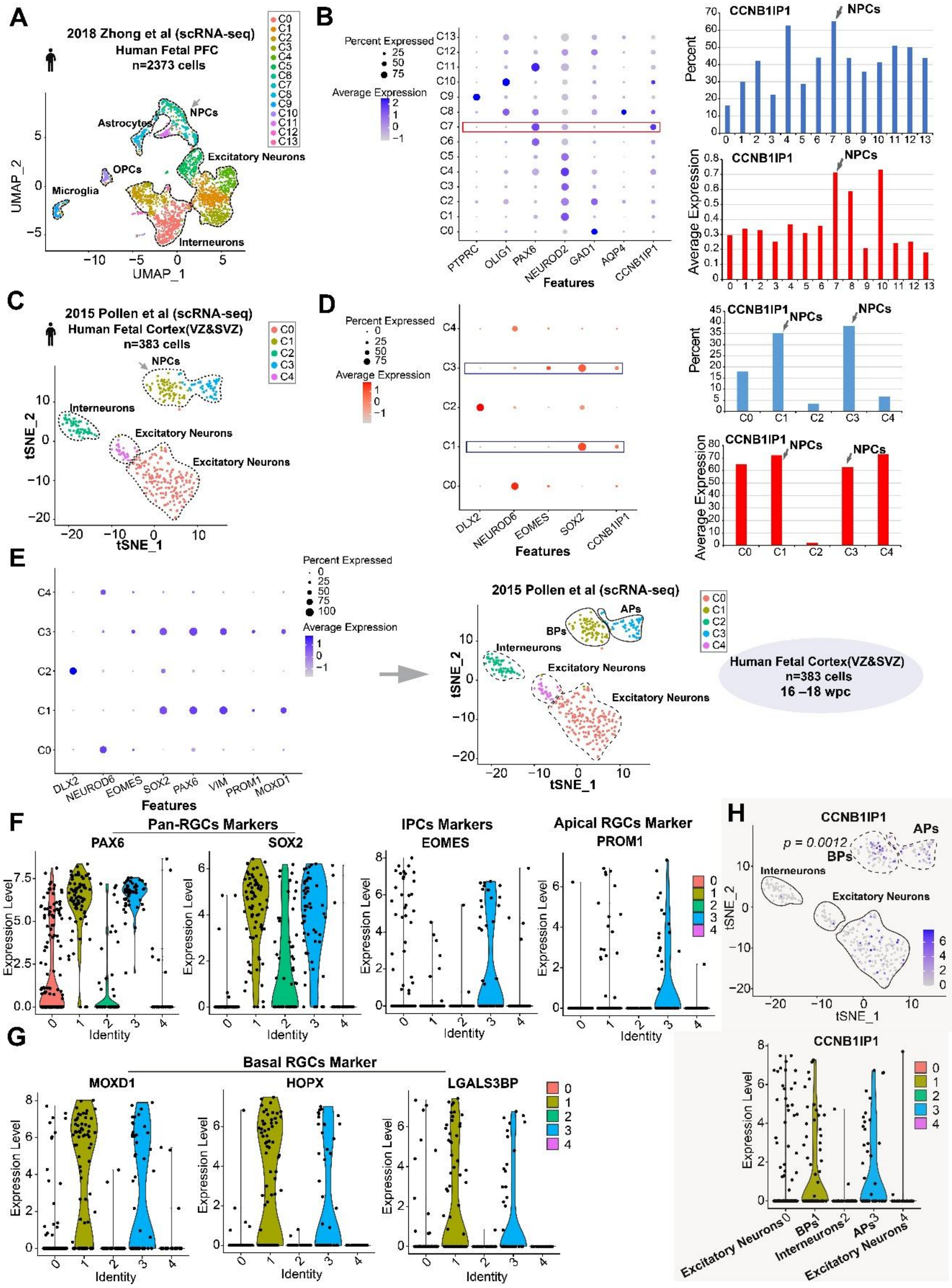

Fig.S2. Identification of human *CCNB1IP1* high expression in NPCs, in which, BPs CCNB1IP1 expression is higher than APs.

(**A**) Uniform manifold approximation and projection (UMAP) plot showing cell types identified in the human fetal prefrontal cortex (PFC).

bioRxiv preprint doi: https://doi.org/10.1101/2024.12.08.627376; this version posted December 9, 2024. The copyright holder for this preprint (which was not certified by peer review) is the author/funder, who has granted bioRxiv a license to display the preprint in perpetuity. It is made available under aCC-BY 4.0 International license.

(**B**) Expression intensity and percentage of *CCNB1IP1* in the identified cell types.

(**C**) A t-distributed stochastic neighbour embedding (t-SNE) plot showing cell types identified from the dissected germinal zones of the human fetal cortex.

(**D**) Expression intensity and percentage of *CCNB1IP1* in the identified cell types.

(**E**) (left panel) Expression intensity and percentage of marker genes in identified cell types; (right panel) a t-SNE plot showing that NPCs cluster3 belongs to APs mixed with EOMES expression and NPCs cluster1 belongs to BPs at human 16-18wpc.

(**F**) Violin plots showing the expression of pan-RGCs markers *PAX6*, *SOX2*, IPC marker *EOMES* and apical RGCs marker *PROM1*.

(**G**)Violin plots showing the basal RGCs markers *MOXD1*, *HOPX*, and *LGALS3BP* expression pattern.

(**H**) Feature and violin plots demonstrating that *CCNB1IP1* expression is higher in BPs than APs at the peak stage of human neurogenesis.

bioRxiv preprint doi: https://doi.org/10.1101/2024.12.08.627376; this version posted December 9, 2024. The copyright holder for this preprint (which was not certified by peer review) is the author/funder, who has granted bioRxiv a license to display the preprint in perpetuity. It is made available under aCC-BY 4.0 International license.

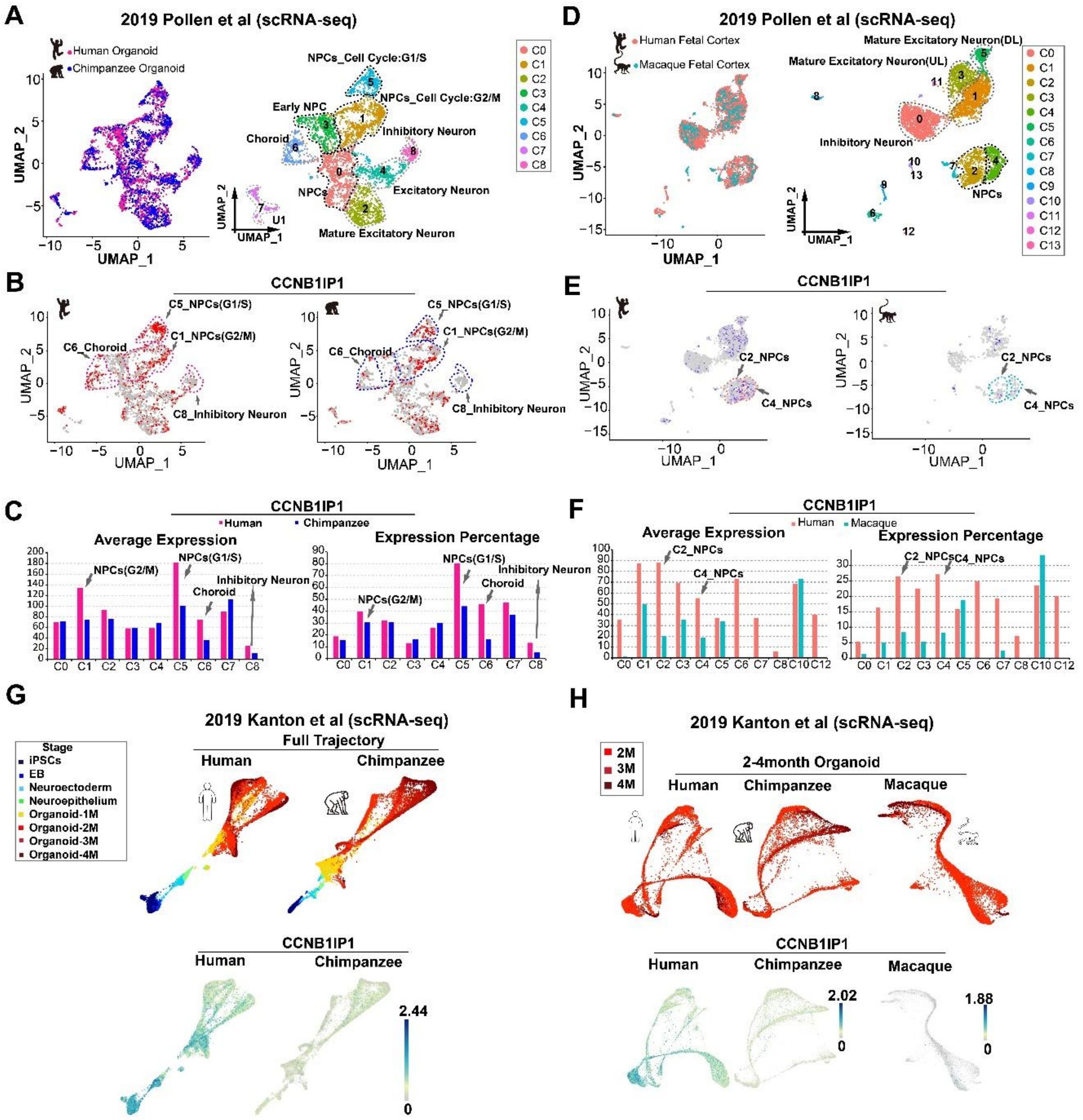

Fig.S3. Human *CCNB1IP1* expression is higher than both chimpanzee and macaque in NPCs.

(**A**) A UMAP plot showing cell types identified in human and chimpanzee organoids (Pollen et al., 2019).

(**B**) UMAP plots showing *CCNB1IP1* expression in human and chimpanzee organoids.

(**C**) Expression intensity and percentage of *CCNB1IP1* in the identified cell types. Arrows indicate identified cell types showing higher expression of *CCNB1IP1* in humans.

bioRxiv preprint doi: https://doi.org/10.1101/2024.12.08.627376; this version posted December 9, 2024. The copyright holder for this preprint (which was not certified by peer review) is the author/funder, who has granted bioRxiv a license to display the preprint in perpetuity. It is made available under aCC-BY 4.0 International license.

(**D**) A UMAP plot showing cell types identified from human and macaque fetal cortices (Pollen et al., 2019).

(**E**) UMAP plots showing *CCNB1IP1* expression in human and macaque fetal cortices.

(**F**) Expression intensity and percentage of *CCNB1IP1* in the identified cell types. Arrows indicate identified cell types showing higher *CCNB1IP1* expression in humans. * Clusters 9, 11, and 3 were excluded from the analysis, owing to zero or one cell in the macaque dataset.

(**G-H**) *CCNB1IP1* expression in human, chimpanzee, and macaque organoids, indicating high human *CCNB1IP1* expression during organoid development. *Single-cell expression data were downloaded from (https://bioinf.eva.mpg.de/shiny/sample-apps/scApeX/).

bioRxiv preprint doi: https://doi.org/10.1101/2024.12.08.627376; this version posted December 9, 2024. The copyright holder for this preprint (which was not certified by peer review) is the author/funder, who has granted bioRxiv a license to display the preprint in perpetuity. It is made available under aCC-BY 4.0 International license.

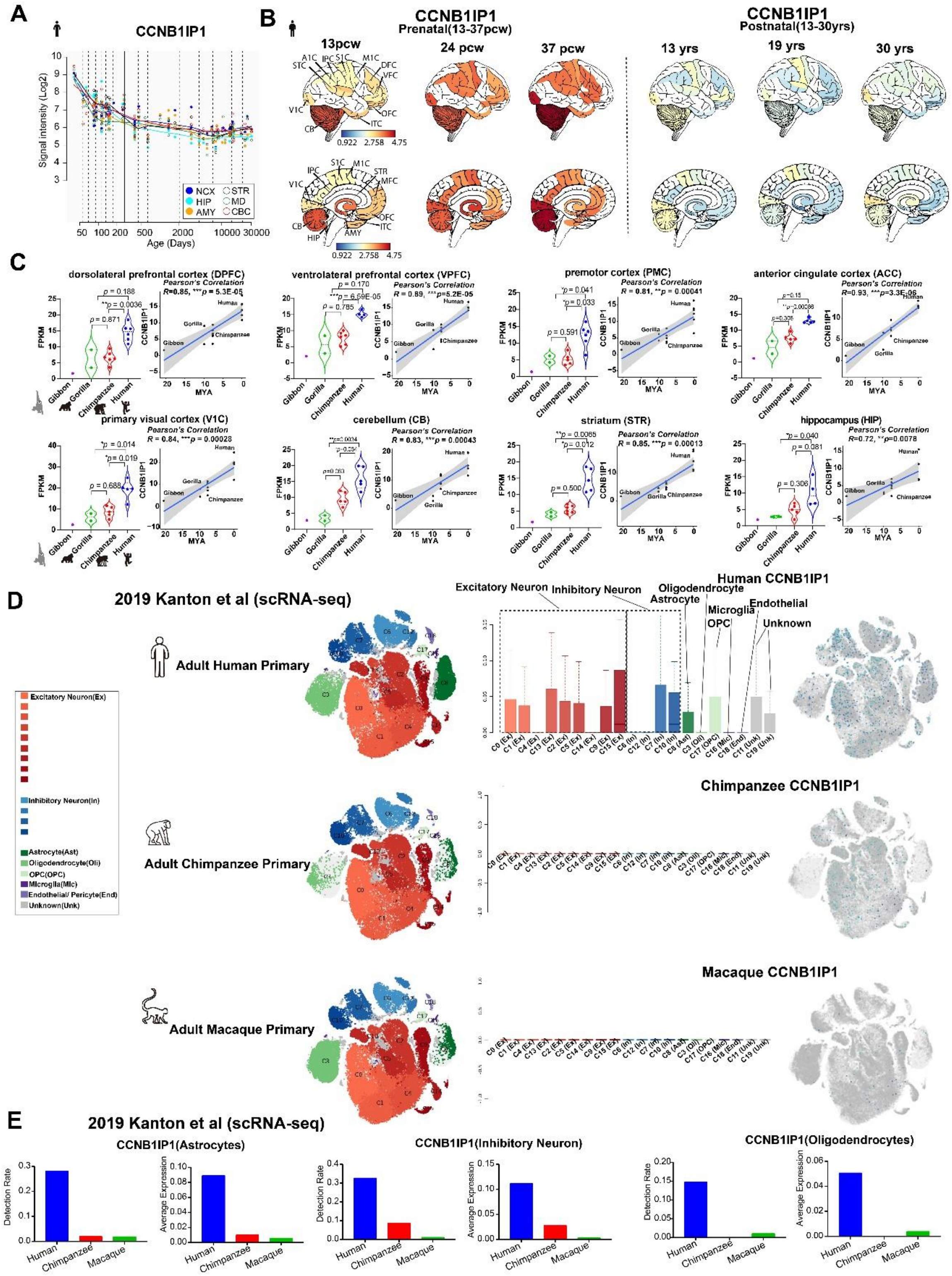

Fig.S4. Expression analysis of *CCNB1IP1* at postnatal stages.

(**A**) Changes in *CCNB1IP1* expression in brain regions, including the neocortex (NCX), hippocampus (Hip), amygdala (AMY), striatum (STR), mediodorsal nucleus of the thalamus (MD), and cerebellar cortex (CBC) during human brain development. The

bioRxiv preprint doi: https://doi.org/10.1101/2024.12.08.627376; this version posted December 9, 2024. The copyright holder for this preprint (which was not certified by peer review) is the author/funder, who has granted bioRxiv a license to display the preprint in perpetuity. It is made available under aCC-BY 4.0 International license.

data were downloaded from the Human Brain Transcriptome Database (http://hbatlas.org/pages/hbtd).

(**B**) Spatial *CCNB1IP1* expression changes at the prenatal and postnatal stages. *RNA sequencing data were downloaded from the Brain Span Atlas of the Developing Human Brain at the Allen Institute.

(**C**) Violin plots and Pearson's correlation analysis of *CCNB1IP1* expression in eight brain regions of humans, chimpanzees, gorillas, and gibbons.

(**D-E**) *CCNB1IP1* expression in human, chimpanzee, and macaque primary brains. *Single-cell expression data were downloaded from (https://bioinf.eva.mpg.de/shiny/sample-apps/scApeX/).

bioRxiv preprint doi: https://doi.org/10.1101/2024.12.08.627376; this version posted December 9, 2024. The copyright holder for this preprint (which was not certified by peer review) is the author/funder, who has granted bioRxiv a license to display the preprint in perpetuity. It is made available under aCC-BY 4.0 International license.

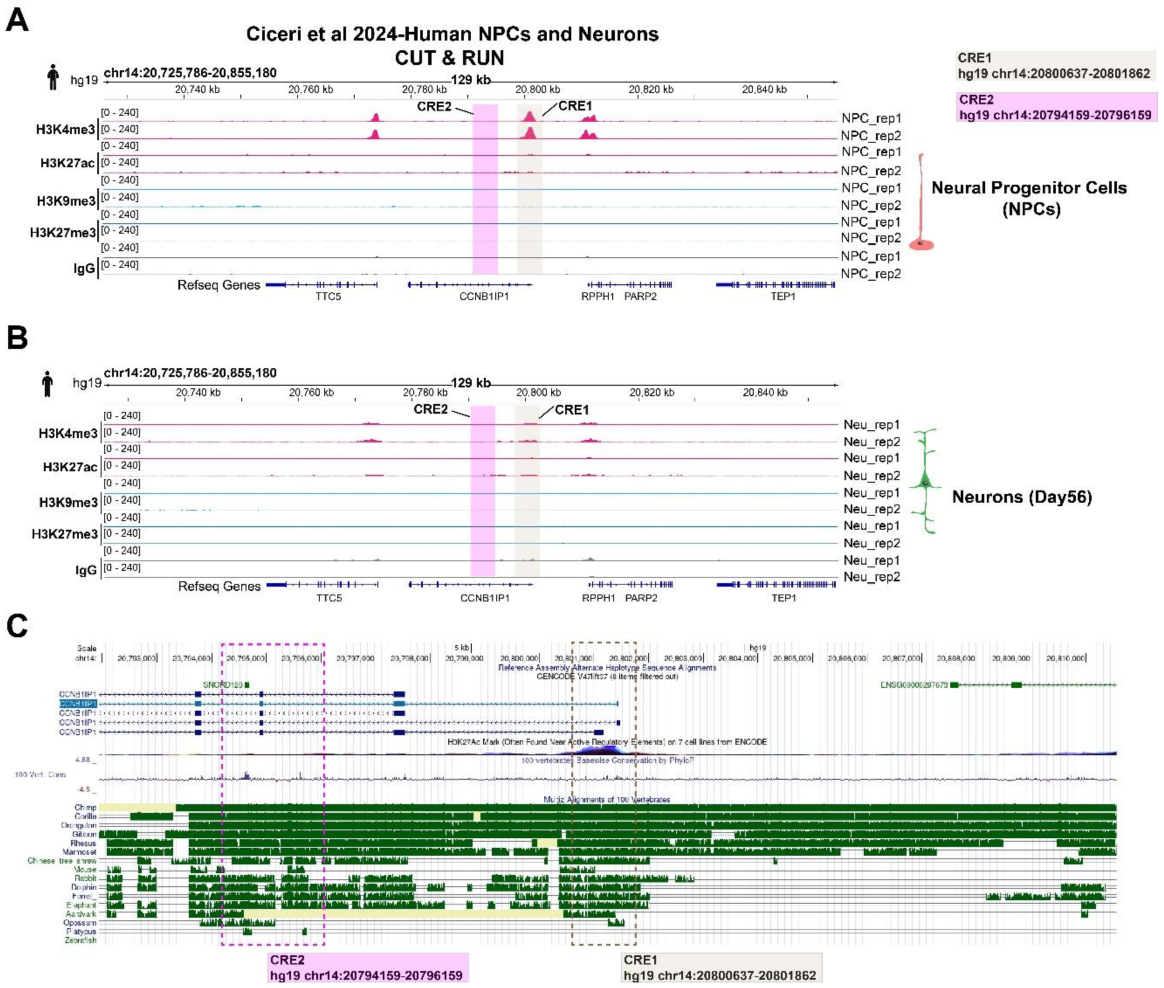

Fig.S5. Histone modification and sequence conservation analysis of the *CCNB1IP1* regulatory elements CRE1 and CRE2.

(**A-B**) Representative tracks of H3K4me3, H3K27ac, H3K9me3, and H3K27me3 at the CCNB1IP1 genomic loci by analyzing published CUT & RUN data in human NPCs and differentiated neurons. *CRE1 and CRE2 masked with square in brown and pink color respectively.

(**C**) Screenshot of CCNB1IP1 genomic loci with multi-species comparisons (green color) using UCSC genome browser. * CRE1 and CRE2 are highlighted in brown and pink square respectively.

bioRxiv preprint doi: https://doi.org/10.1101/2024.12.08.627376; this version posted December 9, 2024. The copyright holder for this preprint (which was not certified by peer review) is the author/funder, who has granted bioRxiv a license to display the preprint in perpetuity. It is made available under aCC-BY 4.0 International license.

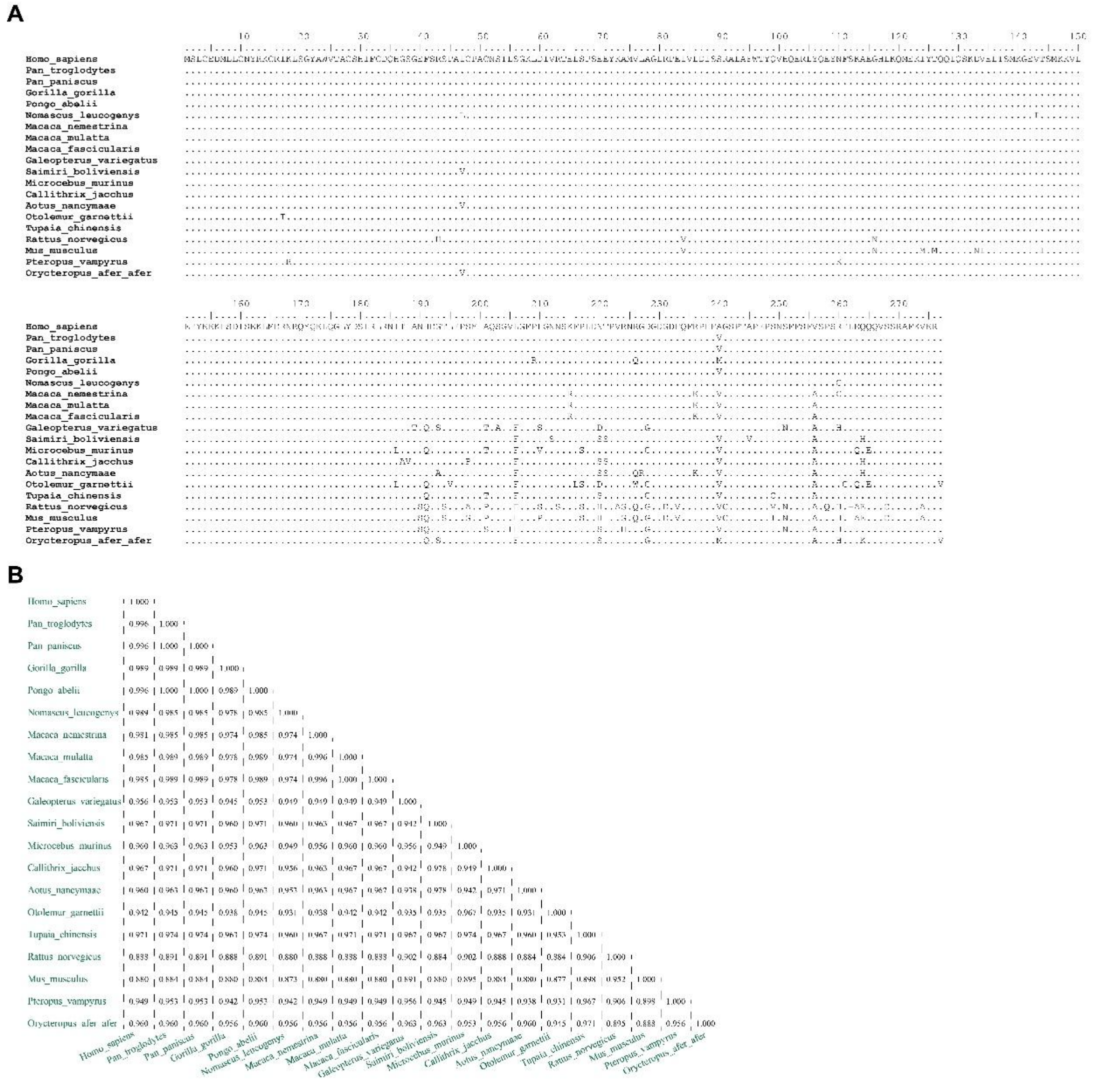

Fig.S6. Multi-species comparison analysis of CCNB1IP1 protein sequence.

(**A**) Amino acid alignments of the CCNB1IP1 protein sequences of 20 mammalian species.

(**B**) Sequence identity matrix results from pairwise comparison of the CCNB1IP1 protein among 20 mammalian species.

bioRxiv preprint doi: https://doi.org/10.1101/2024.12.08.627376; this version posted December 9, 2024. The copyright holder for this preprint (which was not certified by peer review) is the author/funder, who has granted bioRxiv a license to display the preprint in perpetuity. It is made available under aCC-BY 4.0 International license.

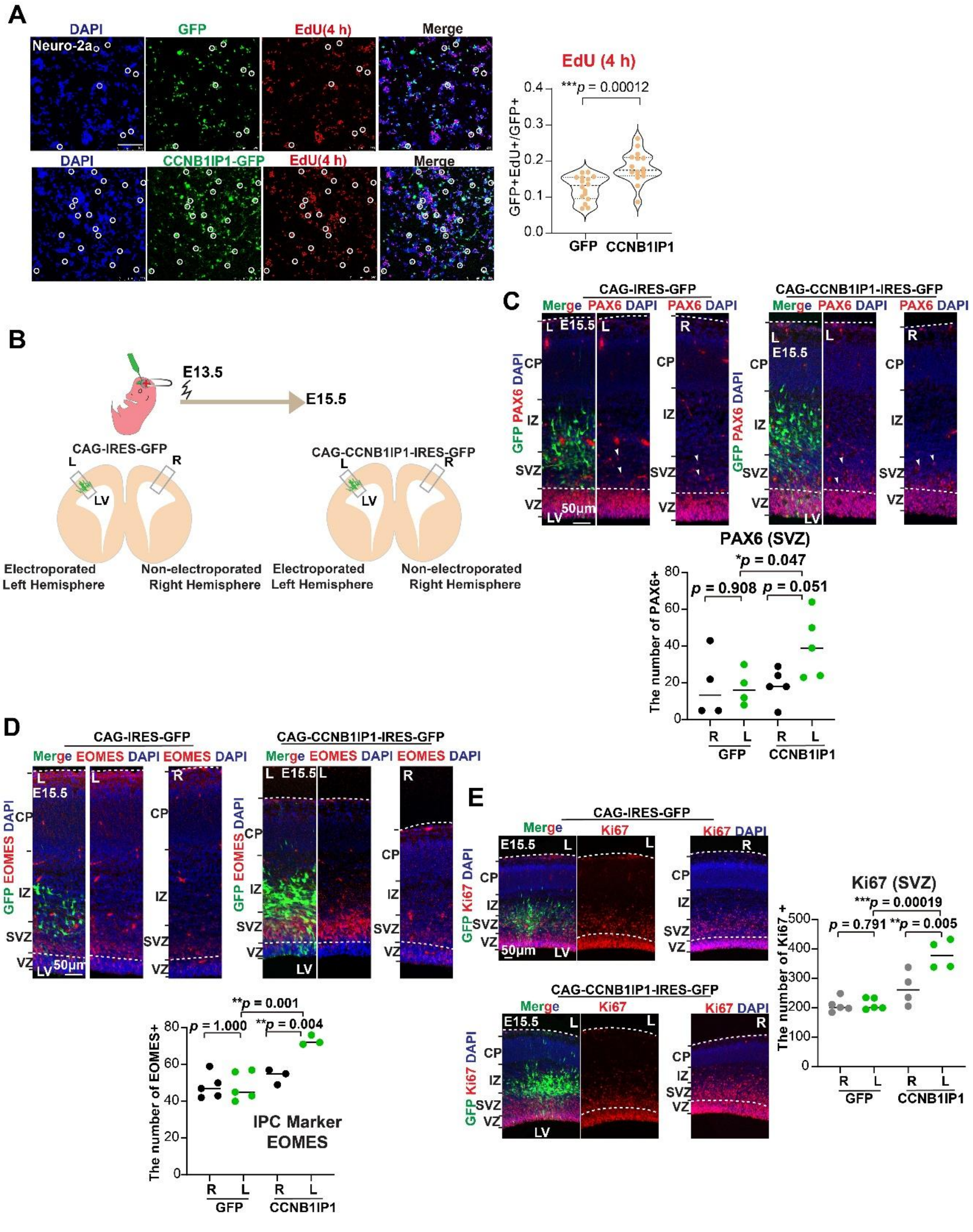

Fig.S7. *CCNB1IP1* promotes the proliferation of BPs.

(**A**) (Top panel) Representative images of cells transfected with CCNB1IP1-GFP and GFP with EdU pulses for 4 h. (Bottom panel) Violin plots of the percentages of $GFP^{+}EdU^{+}$ in GFP^{+} cells. (n=16 vision fields for totally 4 replicates, data represented as mean± SD, *$p < 0.05$, unpaired Student's *t*-test).

bioRxiv preprint doi: https://doi.org/10.1101/2024.12.08.627376; this version posted December 9, 2024. The copyright holder for this preprint (which was not certified by peer review) is the author/funder, who has granted bioRxiv a license to display the preprint in perpetuity. It is made available under aCC-BY 4.0 International license.

(**B**) A schematic diagram of the IUE (E13.5-E15.5) strategy.

(**C**) (Top panel) Immunofluorescence images of the APs marker PAX6 at E15.5 in the mouse embryonic cortex. *Arrows indicate PAX6$^+$ cells. (Bottom panel) Quantification of PAX6$^+$ cells in electroporated and non-electroporated regions, as shown in (a) (n=3-5 embryos from two IUE experiments, data represented as mean, *$p < 0.05$, unpaired Student's t-test).

(**D**) (Top panel) Immunofluorescence images of the IPC marker EOMES at E15.5 in the mouse embryonic cortex. (Bottom panel) Quantification of EOMES$^+$ cells in electroporated and non-electroporated regions, as indicated in (A), (n=3-5 embryos from two IUE experiments, data represented as mean± SD, *$p < 0.05$, **$p < 0.001$, unpaired Student's t-test).

(**E**) (Top panel) Immunofluorescence of cell cycle marker Ki67 after electroporation at E13.5. (Bottom panel) quantification of Ki67$^+$ cells at electroporated and non-electroporated regions (n=4-5 embryos from two IUE experiments, data represented as mean± SD, **$p < 0.001$, ***$p < 0.001$, unpaired Student's t-test).

bioRxiv preprint doi: https://doi.org/10.1101/2024.12.08.627376; this version posted December 9, 2024. The copyright holder for this preprint (which was not certified by peer review) is the author/funder, who has granted bioRxiv a license to display the preprint in perpetuity. It is made available under aCC-BY 4.0 International license.

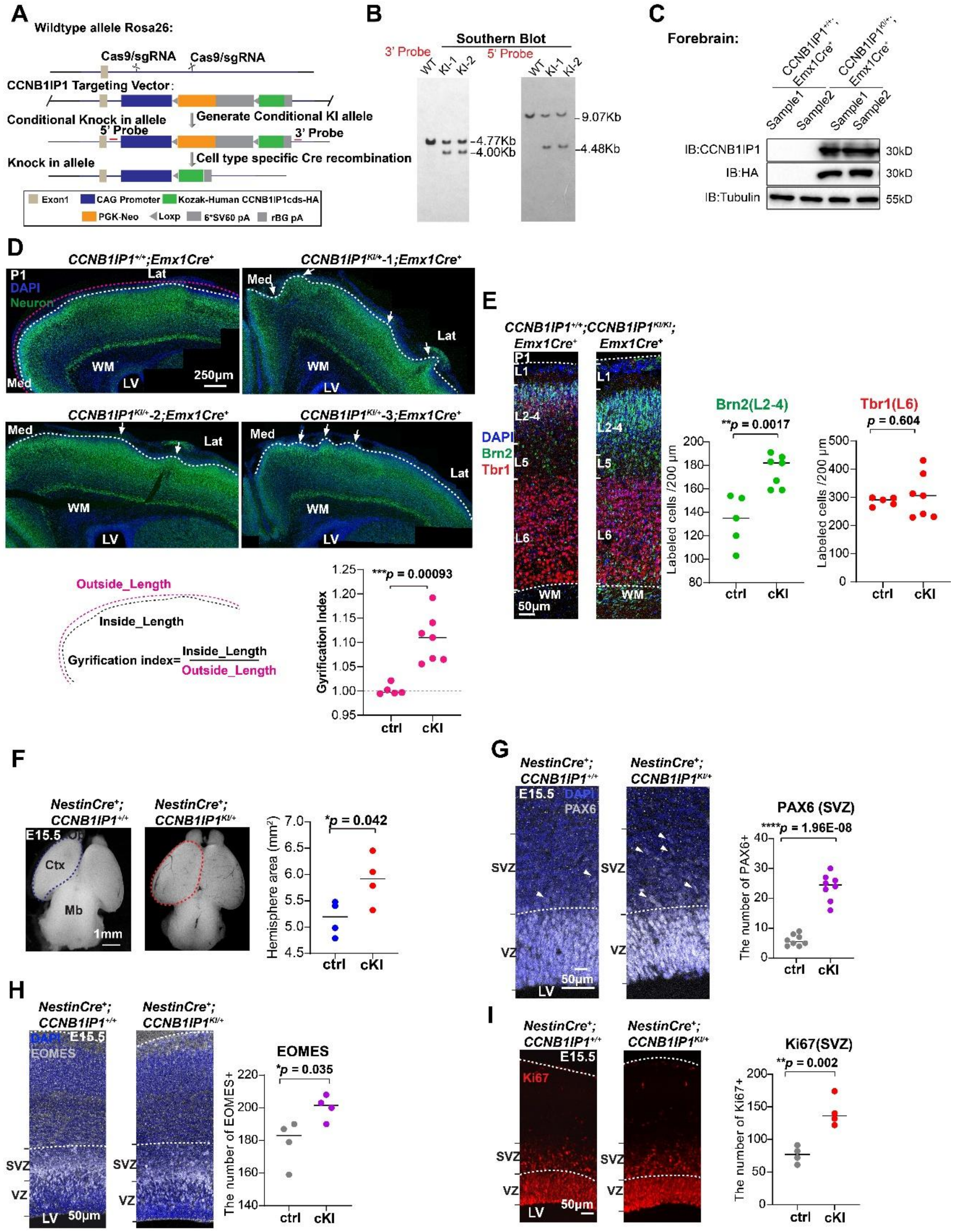

Fig.S8. Generation of human *CCNB1IP1* conditional knock-in mice and cortical folding analysis in cKI.

(**A**) Schematic representation of the generation of human *CCNB1IP1* conditional knock-in mice at the Rosa26 locus using CRISPR-Cas9 technology.

bioRxiv preprint doi: https://doi.org/10.1101/2024.12.08.627376; this version posted December 9, 2024. The copyright holder for this preprint (which was not certified by peer review) is the author/funder, who has granted bioRxiv a license to display the preprint in perpetuity. It is made available under aCC-BY 4.0 International license.

(**B**) A successful knock-in *CCNB1IP1* allele was verified through Southern blotting using 5′ and 3′ probes surrounding the targeting cassette.

(**C**) Western blotting analysis of *Emx1-Cre;CCNB1IP1*$^{KI/+}$ cKI mouse cortex, showing high *CCNB1IP1* expression compared to controls.

(**D**) (Top panel) Representative images of cortical folding (arrows) in cKI$^{Emx1\text{-}Cre}$ mice at P1; (Bottom panel) Schematic representation and quantification of the degree of cortical folding in cKI$^{Emx1\text{-}Cre}$ mice. Each data point represents a sample, 5 controls and 7 cKI from two nests.

(**E**) Immunostaining and quantification of the upper layer marker Brn2 (green) and deeper layer marker Tbr1 (red) in cKI$^{Emx1\text{-}Cre}$ mice, indicating significantly increased upper layer neurons. Each data point represents a sample. 5 controls and 7 cKI from two nests.

(**F**) A dorsal view of the control and cKI$^{Nestin\text{-}cre}$ embryonic brains at E15.5 (left panel) and quantification results showing significant increase of the cortical area in cKI$^{Nestin\text{-}cre}$ mice (right panel). Each data point represents a sample, 4 controls and 4 cKI from one nest.

(**G-I**) Representative images and quantification of PAX6^{+}, EOMES^{+}, and Ki67^{+} cells in cKI$^{Nestin\text{-}cre}$ mice. Each data point represents a sample.

All statistical data are presented as the mean or mean, $*p < 0.05$, $**p < 0.001$, $***p < 0.001$, *n.s*, not significant, as determined using the unpaired Student’s *t*-test.

bioRxiv preprint doi: https://doi.org/10.1101/2024.12.08.627376; this version posted December 9, 2024. The copyright holder for this preprint (which was not certified by peer review) is the author/funder, who has granted bioRxiv a license to display the preprint in perpetuity. It is made available under aCC-BY 4.0 International license.

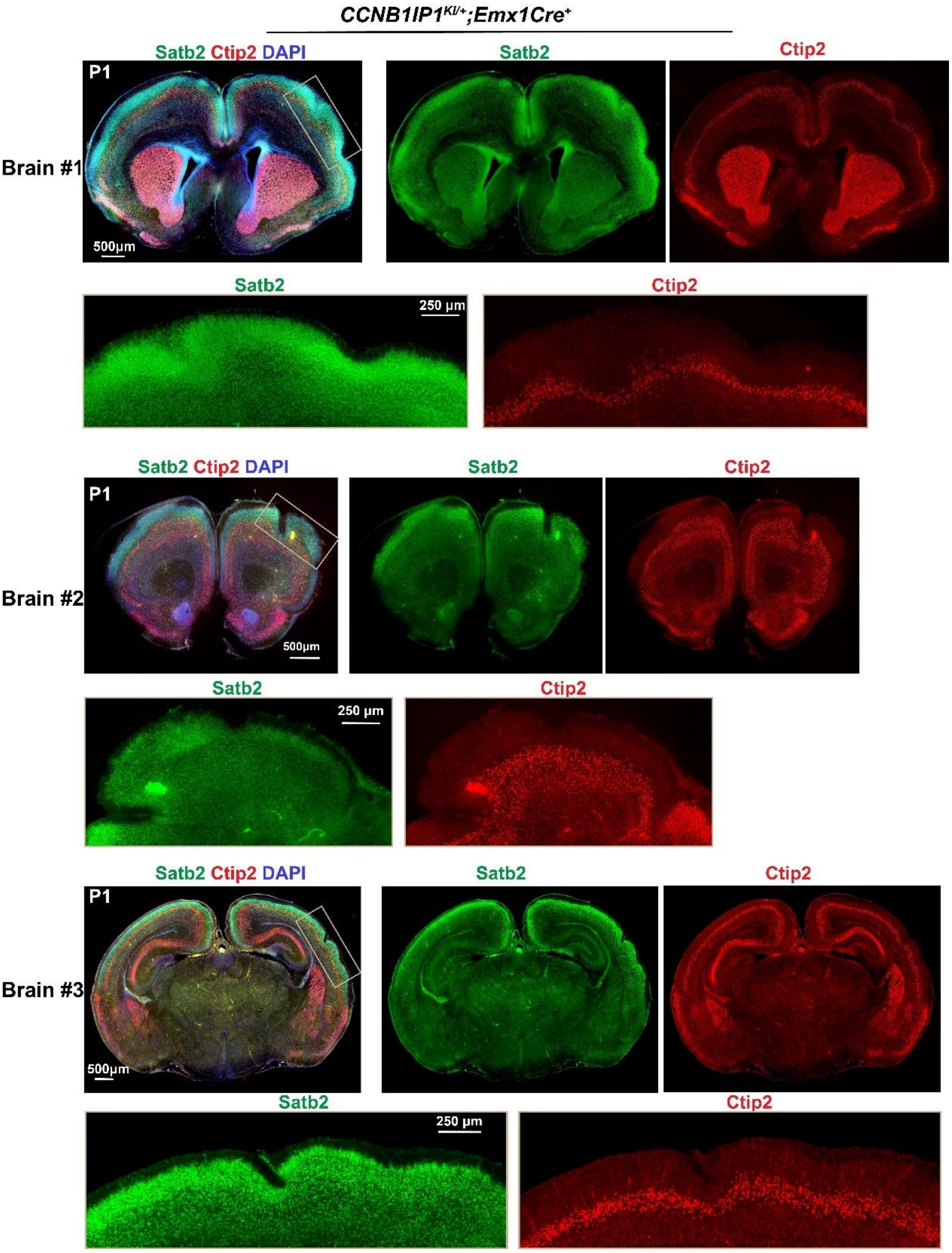

Fig.S9. cortical folding analysis of cKI at P1.

Representative images of cortical layer marker SATB2 (L2-4) and CTIP2 (L6) in three independent cKI mice collected from different nests at P1. Area with white rectangle to be magnified to show the gyri and adjacent sulci structures in the folding locations.
